# Navigation und Robotik – status präsens und zukünftige Implikationen

**DOI:** 10.1007/s00132-023-04468-1

**Published:** 2024-01-31

**Authors:** Günther Maderbacher, Clemens Baier, Matthias Meyer, Dominik Holzapfel, Stefano Pagano, Joachim Grifka, Felix Greimel

**Affiliations:** Orthopädische Klinik, Universität Regensburg, Asklepios Klinikum Bad Abbach, Kaiser-Karl-V.-Allee 3, 93077 Bad Abbach, Deutschland

**Keywords:** Kniegelenk, Untere Extremitäten, Roboterassistierte Chirurgie, Chirurgische Navigationssysteme, Totaler Kniegelenkersatz, Knee joint, Lower limb, Robot-assisted surgery, Surgical navigation systems, Total knee replacement

## Abstract

**Einleitung:**

Sowohl Navigationssysteme als auch die Robotik ermöglichen eine höhere Präzision bei der Implantation eines künstlichen Kniegelenkes. Eine Verbesserung der klinischen Ergebnisse kann dadurch aber nicht erreicht werden. Wir stellten die Hypothese auf, dass es im Rahmen der Implantation einer Knietotalendoprothese zwar zur Rekonstruktion des Alignments in der Koronarebene kommt, durch die variable rotatorische Tibia- sowie variable translatorische Femur- und Tibiakomponentenpositionierung zu einer Veränderung der restlichen Alignmentparameter der unteren Extremität kommt. Diese Parameter könnten jedoch mittels Navigationssystem oder Roboter bestimmt werden und könnten zukünftige Implikationen für diese Systeme darstellen.

**Methoden:**

In 9 gesunden Kniegelenken von fixierten Ganzkörperleichen nach Thiel erfolgte die Bestimmung der Kinematik (Rollback bzw. tibiale Innenrotation sowie tibiale Ab‑/Adduktion) und der Stellung zwischen Femur bzw. Epikondylen und Tibia vor und nach Implantation einer Knietotalendoprothese zwischen 0 und 90° Beugung mithilfe eines Navigationssystems (Knee 2.6, Fa. Brainlab, München, Deutschland).

**Ergebnisse:**

Nach endoprothetischer Versorgung kam es zu keiner Veränderung des natürlichen koronaren Alignment. In Streckung und den frühen Beugegraden zeigte sich die Rotationsstellung des Femurs gegenüber der Tibia verändert. Dies führte auch zu einer veränderten Positionierung des Epicondylus medialis und lateralis in Relation zur Tibia: Während beide Epikondylen nach endoprothetischer Versorgung in Relation zur Tibia lateraler positioniert waren, war der Epicondylus lateralis bis 20° Beugung signifikant dorsaler gelegen.

**Diskussion:**

Nach endoprothetischer Versorgung eines Kniegelenkes in etablierter Technik kam es zu einer guten Rekonstruktion des koronaren Alignments bei gleichzeitiger Veränderung des Alignments sowohl in rotatorischer als auch translatorischer Richtung zwischen Femur und Tibia. Mittels Navigation aber auch Robotik wären wir in der Lage, sämtliche Alignmentparameter zu quantifizieren und könnten eine Ausrichtung der Komponenten bzw. eine Rekonstruktion des Gesamtalignments in allen sechs Freiheitsgraden erzielen. Womöglich wären wir dadurch in der Lage, auch einen klinischen Vorteil zu erzielen bzw. es könnten die Standzeiten noch weiter erhöht werden.

**Graphic abstract:**

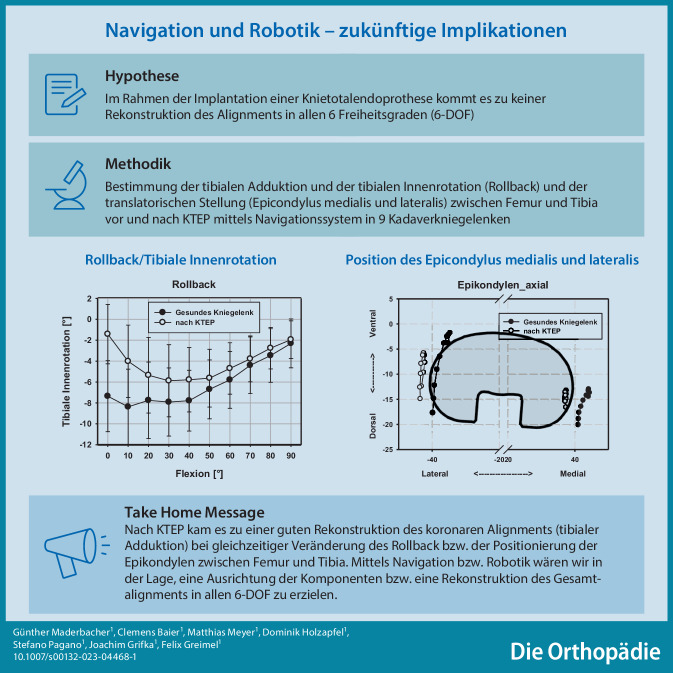

## Abstract (en)

**Introduction:**

Both navigation systems and robotics enable greater precision in the implantation of an artificial knee joint. However, they do not improve clinical outcomes. We hypothesized that although implantation of a total knee arthroplasty results in reconstruction of the alignment in the coronal plane, the variable rotational tibial and variable translational femoral and tibial component positioning lead to a change in the remaining alignment parameters of the lower extremity. However, these parameters could be determined using a navigation system or robot and could represent future implications for these systems.

**Methods:**

The kinematics and the position between femur and tibia before and after implantation of a total knee arthroplasty were determined using a navigation system in nine healthy knee joints of Thiel-fixed whole-body cadavers.

**Results:**

After arthroplasty, there was no change in the natural coronal alignment. In extension and the early degrees of flexion, the rotational position of the femur relative to the tibia was altered. This also led to a change in the positioning of the medial and lateral epicondyle in relation to the tibia; while both epicondyles were positioned more laterally in relation to the tibia after arthroplasty, the lateral epicondyle was significantly more lateral in relation to the tibia up to 20° of flexion.

**Discussion:**

Following arthroplasty of a knee joint using the established technique, a good reconstruction of the coronal alignment was achieved with simultaneous changes in the alignment in both the rotational and translational directions between the femur and tibia. Using navigation as well as robotics, we would be able to quantify all alignment parameters and could achieve an alignment of the components or a reconstruction of the overall alignment in all six degrees of freedom. We might also be able to achieve a clinical advantage or increase the service life even further.

Die Endoprothetik des Kniegelenkes zählt zweifelsohne zu den erfolgreichsten Verfahren in der Orthopädie. Grundsätzliche Techniken, die bis heute Gültigkeit besitzen, sowie Implantate wurden bereits vor Jahrzehnten entwickelt. Damit kann mit einer sehr hohen Zuverlässigkeit eine adäquate Patientenzufriedenheit erreicht werden. Kontinuierlich werden seitdem Anstrengungen unternommen die Erlebnisqualität zu steigern.

## Einführung

Für eine lange Überlebensdauer wurde eine gerade Beinachse propagiert, um eine gleichmäßige Belastung des Implantats bzw. des Implantat-Knochen-Interface zu gewährleisten. Trotz immenser Anstrengungen in der Instrumentenentwicklung zeigte sich, dass ein gewünschtes Ziel einer geraden Beinachse mit einer Abweichung ± 3° nur in 8–9 von 10 Fällen erreicht werden kann [1].

Mit der Entwicklung computergestützter Assistenzsysteme (CAS) in der Orthopädie konnte um die Jahrtausendwende ein entscheidender Schritt in Richtung höherer Präzision in der Implantation künstlicher Gelenke getätigt werden.

Über eine Detektionseinheit sowie Referenzmarker, die fest mit dem Femur wie der Tibia verankert werden, kann im Falle von CT-basierten Systemen eine vorab angefertigte CT des Kniegelenks bzw. der betroffenen Extremität gematcht werden, oder im Falle von CT-freien Systemen die individuelle Oberflächen- und Achsgeometrie der unteren Extremität bestimmt werden. Über das Navigationssystem können in weiterer Folge die für das Implantat notwendige Schnittblöcke dreidimensional mit einer Genauigkeit von ca. 1° bzw. 1 mm angebracht werden [2, 3]. Nach erfolgter Resektion kann die Verifikation der tatsächlich erfolgten Schnittebene erfolgen.

Mithilfe der Navigationstechnik konnten wir, wie auch viele andere Studiengruppen, eine deutlich höhere Präzision hinsichtlich der Rekonstruktion einer geraden Beinachse nachweisen, als es mit mechanischen Ausrichtinstrumentarien möglich ist. Eine Verbesserung der klinischen Ergebnisse hingegen konnte mit den Navigationssystemen trotz erhöhter Präzision nicht erreicht werden. Auch die kurz- und mittelfristigen Standzeiten von Kniegelenkendoprothesen konnte durch die Navigation nicht verbessert werden.

Aufgrund der hohen Anschaffungs- und Betriebskosten von Navigationssystemen, der deutlich verlängerten Operationsdauer von etwa 15 min selbst in einem spezialisierten Setting und der komplexen Operationstechnik bei gleichzeitig nicht nachweisbarem klinischem Vorteil kam es zu einem rückläufigen Einsatz von navigationsgestützten Knieendoprothesenimplantationen.

Erst Veröffentlichungen des australischen Prothesenregisters ließen vor wenigen Jahren die Navigation wieder in das Licht der Öffentlichkeit rücken [4]: Es konnte gezeigt werden, dass sich die kumulative Revisionsrate nach KTEP-Implantation bei Patienten mit einem Alter von 65 Jahren und jünger, wenn ein Navigationssystem verwendet wurde, reduzierte (7,8 % vs. 6,3 %). Auch in unserem Kollektiv konnte in der Gruppe der navigierten KTEP eine geringere Revisionsrate nach durchschnittlich 10 Jahren gefunden werden [5].

In den vergangenen Jahren wurde die computerassistierte Chirurgie in der Endoprothetik insbesondere über die Industrie wieder deutlich forciert. Dabei wurde mit der sogenannten Roboterchirurgie ein Thema aufgegriffen, das aufgrund der Historie – Stichwort Robodoc® (Think Surgical, USA) – zumindest in der älteren orthopädischen Generation nicht ganz ohne Vorurteile behaftet ist.

Naturgemäß werden hier von verschiedenen Herstellern unterschiedliche Modelle angeboten, die sich im Grundsatz allesamt ähneln:

Als Basis dient ein Computersystem mit einer Navigationseinheit. Dieses wird entsprechend der oben angeführten Navigationssysteme mit CT-Daten gespeist oder es erfolgt die intraoperative Bestimmung der Anatomie über Referenzmarker.

Neu hinzu kommt nun das Vorhandensein eines Roboterarms. Mithilfe des Computersystems kann der am Roboterarm befestigte Schnittblock dreidimensional in Echtzeit ausgerichtet werden oder das Sägeblatt/die Fräse wird am Roboterarm befestigt und eine Resektion erfolgt direkt über den Roboterarm. Aufgrund der zugrundeliegenden Technik lässt sich eine der Navigation entsprechende sehr hohe Präzision erreichen.

Einen zusätzlichen Schub haben computerassistierte Verfahren und hier insbesondere die Robotik zuletzt aufgrund „moderner“ Alignmentphilosophien erfahren. Unter dem Überbegriff „kinematisches Alignment“ wurde über die letzten Jahre insbesondere durch Stephen Howell ein neues Konzept zur Implantatpositionierung entwickelt. Als Basis dient die komplette Rekonstruktion der ursprünglichen Femuranatomie, wobei distal und dorsal femoral medial und lateral annähernd gleich viel Knochen reseziert wird. Hierbei wird bewusst auf eine Berücksichtigung von Achsen verzichtet, was per se den gewohnten Einsatz von Ausrichtinstrumenten überflüssig macht und sich im Wesentlichen auf Messungen der Resektionen mit der Schublehre beschränkt [6].

Der Einsatz von Robotik für kinematische Alignmentphilosophien scheint daher auf den ersten Blick sogar ein Widerspruch zu sein. Zuletzt wurden verschiedene neue Alignmentkonzepte mit individuellen Abwandlungen des klassischen „kinematic alignment“ propagiert. Beim sogenannten „restricted kinematic alignment“ erfolgt per se eine anatomische Resektion des Femurs sowie der Tibia. Wird dabei möglicherweise der vorgegebene Korridor bzw. die Grenze hinsichtlich einer Varus- oder Valgusausrichtung des Beines bzw. der einzelnen Komponenten verlassen, werden die Resektionen adaptiert. Um während der Operation den angestrebten Korridor nicht zu verlassen, kommen Navigationssysteme oder Roboter zum Einsatz. Wissenschaftliche Auswertungen, insbesondere mittel- und langfristige Ergebnisse, fehlen vielfach. Der derzeitige teilweise unkritische Umgang wirkt aus wissenschaftlicher Sicht manchmal befremdlich.

**Figure 1:**
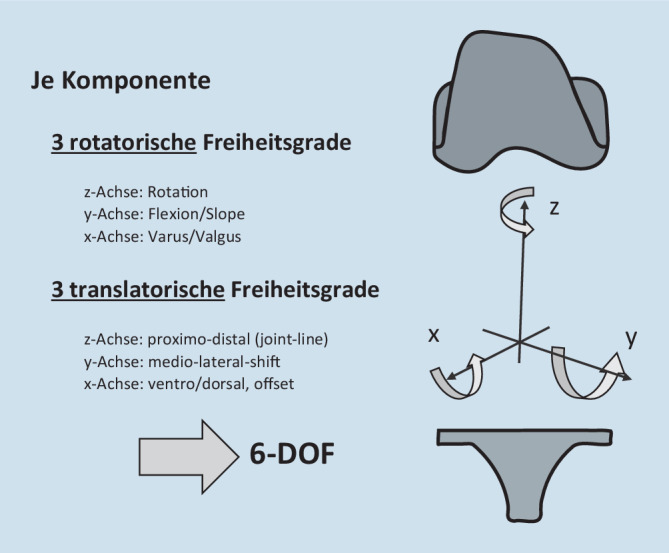

Aktuelle Alignmentphilosophien und wissenschaftliche Diskussionen zielen überwiegend auf das Komponentenalignment in der Koronarebene ab. Sowohl die Femur- wie auch die Tibiakomponente können entsprechend einem dreidimensionalen Koordinatensystem aber in jeweils drei translatorischen (entlang der x‑, y‑ und z‑Achse) und drei rotatorischen (um die x‑, y‑ und z‑Achse) Freiheitsgraden eingesetzt werden [7]: Die Femurkomponentenplatzierung kann rotatorisch hinsichtlich des Varus/Valgus, der Flexion/Extension und der Außen- und Innenrotation adaptiert werden. Translatorisch kann die Komponente über das Ausmaß der distalen Resektion proximaler oder distaler eingesetzt werden, ventraler oder dorsaler implantiert werden sowie die Komponente auch nach medial oder lateral versetzt eingebracht werden. Die Tibiakomponente kann hinsichtlich der rotatorischen Freiheitsgrade ebenfalls in unterschiedlichem Varus/Valgus-Winkel, mit unterschiedlichem Slope bzw. Außen- und Innenrotation eingebracht werden. Je nach Ausmaß der Resektionshöhe kann translatorisch betrachtet die Komponente proximaler oder distaler, ventraler oder dorsaler sowie ebenfalls medial und lateral translatiert eingebracht werden (Abb. 1).

Üblicherweise ist es dem Operateur aktuell nicht möglich, sämtliche Freiheitsgrade einer Endoprothese aktiv zu beeinflussen, weil gerade für translatorische Parameter mit der Ausnahme der distalen Femur- und proximalen Tibiaresektion intraoperativ nur ungenügend Informationen vorliegen, aber auch adäquate Instrumentarien zur Umsetzung fehlen [8, 9].

Wir stellten die Hypothese auf, dass es im Rahmen der Implantation einer KTEP zwar zur Rekonstruktion des Alignment in der Koronarebene kommt, durch die variable rotatorische Tibia sowie variable translatorische Femur- und Tibiakomponentenpositionierung zu einer Veränderung der restlichen Alignmentparameter der unteren Extremität kommt. Diese Parameter könnten jedoch mittels Navigationssystem oder Roboter bestimmt werden und könnten zukünftige Implikationen für diese Systeme darstellen.

## Material und Methoden

Für die vorliegende Studie wurden 9 gesunde Kniegelenke von fixierten Ganzkörperleichen nach Thiel untersucht. Für die Implantation der KTEP sowie die Untersuchung der Kniegelenke wurde ein handelsübliches Navigationssystem verwendet (Knee 2.6, Brainlab, Feldkirchen, Deutschland) [10].

### Operationstechnik

Nach einer medianen Hautinzision erfolgte eine parapatellare Arthrotomie ohne die Kreuzbänder und Menisken zu verletzen. Über Schanz-Schrauben wurden sowohl tibial als auch femoral optische Referenzmarker montiert und danach notwendige Landmarken registriert. Für das Femur: Hüftkopfzentrum, Kniegelenksmittelpunkt, Oberfläche der medialen und lateralen Femurkondyle, Epicondylus medialis und lateralis und Whiteside-Linie. Für die Tibia: Malleoli medialis und lateralis, Eminentia intercondylaris, Akagi-Linie [11] und artikulierende Oberfläche des medialen und lateralen Tibiaplateaus. Hernach wurde die Kapsel anatomisch verschlossen, das Kniegelenk mittels Motorschiene passiv zwischen 0 und 90° gebeugt und dabei die Kinematik des Kniegelenkes bzw. der unteren Extremität über das Navigationssystem zweimalig aufgezeichnet.

In einem weiteren Schritt wurde navigationsgestützt eine KTEP eingesetzt (DePuy Synthes PFC Sigma CR, fixed bearing, DePuy, Warsaw, IN, USA): Menisken und das vordere Kreuzband wurden reseziert. Der Tibiaschnitt erfolgte koronar senkrecht zur mechanischen Achse und 4° Slope mit einer Resektion von 8 mm entsprechend der Implantatdicke. Der distale Femurschnitt wurde senkrecht zur mechanischen Femurachse mit 4° Flexion durchgeführt. Die Femurkomponente wurde 3° nach außen rotiert. Es wurden keine Bandreleases durchgeführt. Die Tibiakomponente wurde in Bezug zur Akagi-Linie in leichter Außenrotation eingesetzt. Hinsichtlich der mediolateralen Ausrichtung wurden sowohl die femorale als auch die tibiale Komponente zentral ausgerichtet, sodass sie den Knochen ohne Überhang abdecken. Die Probekomponenten wurden mit Schrauben und Pins temporär fixiert. Nach dem anatomischen Verschluss der Kapsel wurde wiederum die Kinematik der Kniegelenke/unteren Gliedmaßen zwischen 0° und 90° Beugung innerhalb von zwei Bewegungszyklen aufgezeichnet. Alle Operationen wurden von einem erfahrenen Chirurgen durchgeführt (> 500 navigierte KTEP).

### Kalkulationen

Für die Berechnungen wurde ein anatomiebasiertes Koordinatensystem verwendet [12], das eine tibiale und femorale Matrix definiert. Für die tibiale Matrix wurde die mechanische Tibiaachse (Verbindungslinie zwischen Eminentia intercondylaris und dem Zentrum des Talus) als z‑Achse (zt) definiert. Die Akagi-Linie, die die z‑Achse schneidet, wurde als y‑Achse (yt) definiert. Die x‑Achse (xt) wurde über das Kreuzprodukt der beiden anderen Achsen berechnet. In der Femurmatrix wurde die z‑Achse (zf) ebenfalls als mechanische Femurachse (Verbindung zwischen dem Hüftkopfzentrum und dem distalen Femurzentrum) definiert. Eine Linie, die den medialen und lateralen Epikondylus verbindet und die z‑Achse schneidet, wurde als x‑Achse (xf) definiert. Die y‑Achse (yf) wurde durch das Kreuzprodukt der ersten beiden Achsen berechnet. Die Bewegung im Kniegelenk wird durch ein drittes Koordinatensystem (xk, yk, zk) ausgedrückt, in dem die anatomischen Achsen des tibialen und femoralen Koordinatensystems verwendet werden: Die tibiale Innen- und Außenrotation wird um eine Achse (zk) definiert, die der mechanischen Achse (zt) des Schienbeins entspricht. Beugung und Streckung werden um eine Achse (xk) definiert, die der transepikondylären Achse des distalen Femurs entspricht (xf) entspricht. Durch die Berechnung des Kreuzprodukts zwischen der mechanischen Femurachse (zf) und der transepikondylären Achse (xf) wird eine dritte gleitende Achse (yk) bestimmt, die die Abduktion und Adduktion beschreibt. Für die Translation des Gelenks werden die gleichen Achsen verwendet, während die mediolaterale Verschiebung entlang der xk, die proximodistale Verschiebung entlang der zk und die ventrodorsale Verschiebung entlang der yk erfolgt.

Zur Quantifizierung der relativen Translation zwischen Femur und Tibia wurde die Position des Epicondylus medialis und lateralis vor und nach endoprothetischer Versorgung herangezogen [13].

Mittels einer Transformationsmatrix wird die femorale Matrix in die tibiale Matrix transformiert, indem eine homogene Matrix gebildet wird, die 3 × 3 Parameter für die Rotation (a, b, c, e, f, g, i, j, k) und drei Parameter für die Translation (d, h, l) kombiniert.

### Statistische Methoden

Kontinuierliche Variablen werden als Mittelwert und der Standardabweichung dargestellt. Für den Vergleich der tibiofemoralen Kinematik (Ab‑/Adduktion sowie tibiale Innenrotation; Position der Epicondyli mediales und laterales) vor und nach KTEP wurde ein gepaarter t‑Test durchgeführt. Ein zweiseitiger *p*-Wert von ≤ 0,05 wurde als statistisch signifikant gewertet. Es erfolgte eine Post-hoc-power-Analyse. Die Auswertungen erfolgten mittels SPSS 21.0.0 (IBM, Armonk, NY, USA), die Grafiken wurden mit SigmaPlot 11.0 (Systat Software, Frankfurt, Deutschland) erstellt. Laut unserer Ethikkommission war für die Durchführung der Studie kein Votum erforderlich.

## Ergebnisse

### Rotatorische Parameter

**Figure 2:**
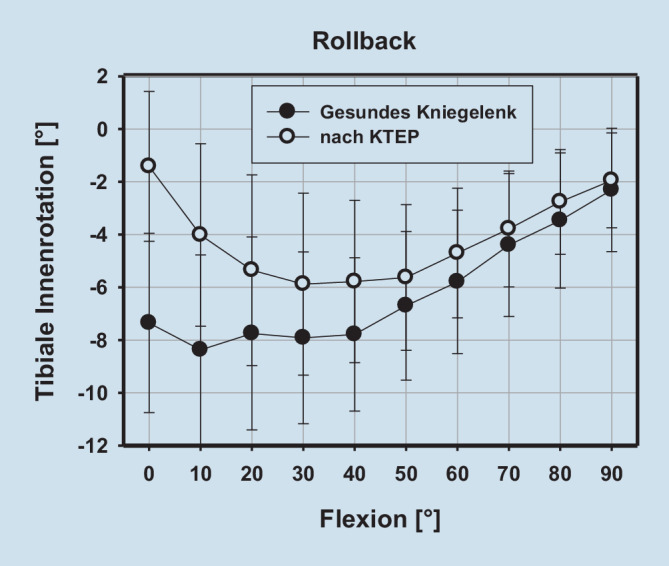

Im untersuchten Kollektiv zeigen gesunde Kniegelenke eine bekannte tibiale Innenrotation (bzw. Rollback) sowie eine tibiale Adduktion bei zunehmender Beugung. Während sich die tibiale Adduktion (koronares Alignment) nach KTEP-Implantation nicht ändert, zeigt sich nach KTEP-Implantation eine signifikant vermehrte tibiale Innenrotation in voller Streckung und 10° Beugung (Tab. 1 und Abb. 2 und 3).

**Table 1:** 

	Rollback			Ab‑/Adduktion		
*Flexion (°)*	*MD*	*SD*	*p‑Wert*	*MD*	*SD*	*p‑Wert*
0	−5,936	5,816	**0,016**	−0,322	1,567	0,554
10	−4,356	4,119	**0,013**	−0,411	1,419	0,41
20	−2,398	4,191	0,124	0,0444	1,339	0,923
30	−2,036	4,418	0,204	0,233	1,29	0,602
40	−2,004	4,533	0,221	0,444	1,234	0,311
50	−1,069	5,461	0,573	0,489	1,418	0,331
60	−1,093	4,951	0,527	0,422	1,806	0,503
70	−0,611	4,883	0,717	0,144	2,192	0,848
80	−0,704	3,985	0,61	0,167	2,489	0,846
90	−0,36	3,197	0,744	0,133	2,949	0,895

Fette Zahlen zeigen einen signifikanten Unterschied (*p* < 0,05)

**Figure 3:**
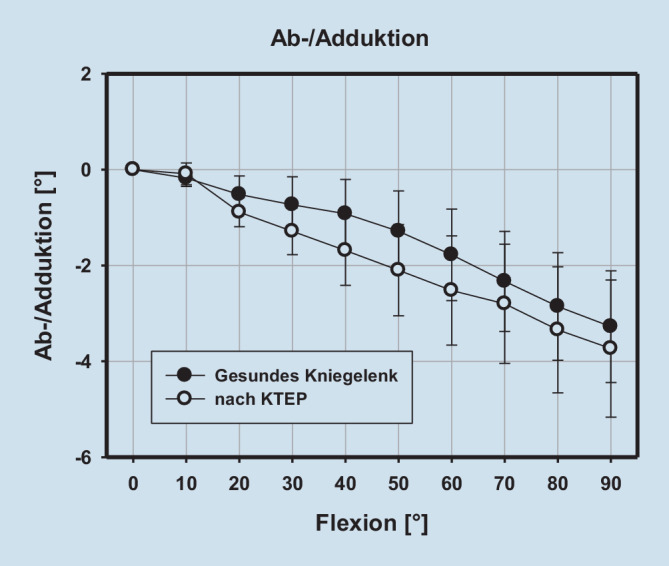

### Translatorische Parameter

**Figure 4:**
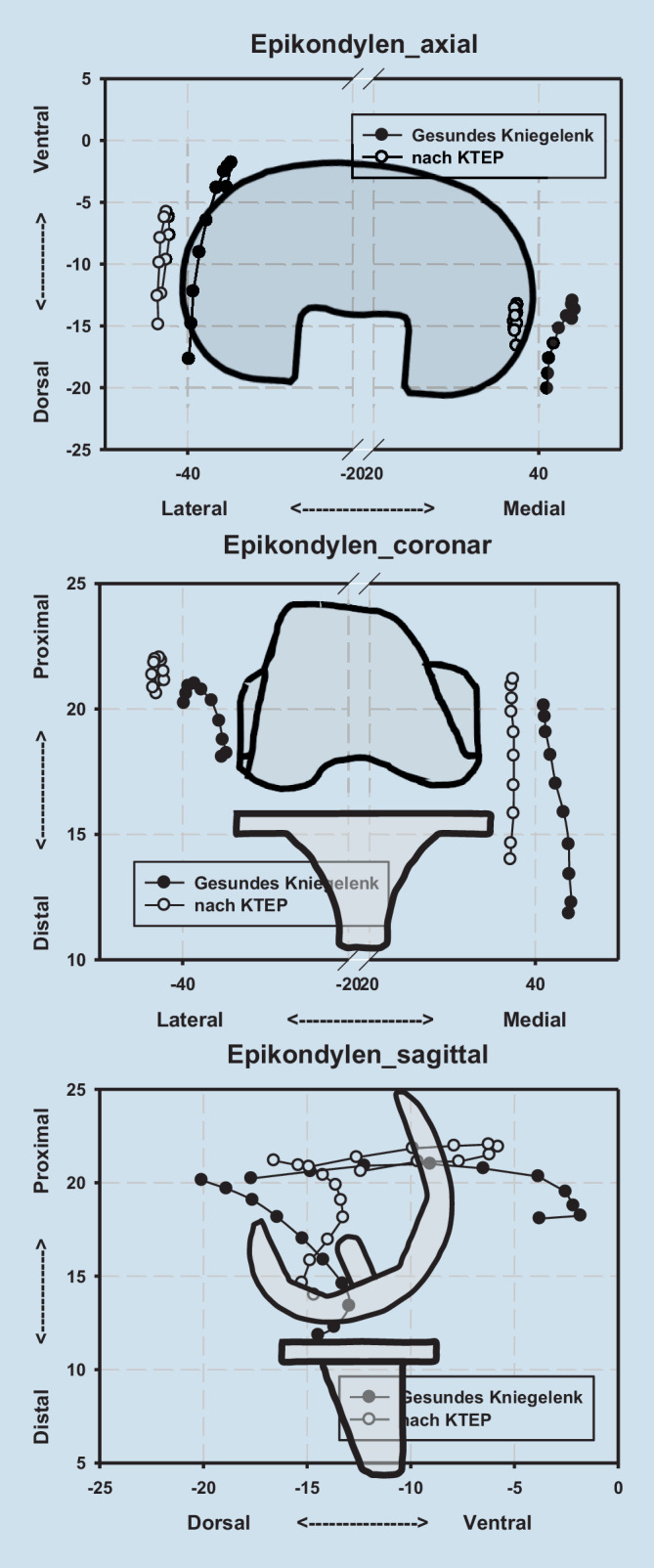

Nach Implantation einer KTEP fand sich eine signifikante Lateraltranslation des medialen wie lateralen Epikondylus zwischen 0 und 80° Beugung. Für den Epicondylus medialis zeigt sich zwischen 40 und 80° Beugung eine leichte Proximalisierung. Der Epicondylus lateralis hingegen zeigt zwischen 0 und 20° Beugung in Bezug auf die Tibia zusätzlich eine Translation nach dorsal (Tab. 2 und 3 sowie Abb. 4).

**Table 2:** 

	Mediolateral			Ventrodorsal			Proximodistal		
*Flexion (°)*	*MD*	*SD*	*p‑Wert*	*MD*	*SD*	*p‑Wert*	*MD*	*SD*	*p‑Wert*
0	7,042	4,174	**<** **0,001**	0,202	6,205	0,924	−2,157	3,807	0,128
10	7,284	4,354	**0,001**	1,555	4,723	0,352	−2,369	3,88	0,104
20	6,728	4,401	**0,002**	1,885	3,689	0,164	−2,426	3,523	0,073
30	6,626	4,622	**0,003**	0,692	4,066	0,623	−2,348	3,238	0,061
40	6,005	4,571	**0,004**	−0,961	4,823	0,567	−2,255	2,72	**0,038**
50	5,071	4,542	**0,01**	−1,859	5,171	0,312	−2,049	2,341	**0,03**
60	4,711	4,518	**0,014**	−2,825	5,348	0,152	−1,721	1,725	**0,017**
70	4,058	4,665	**0,031**	−3,399	5,333	0,092	−1,34	1,371	**0,019**
80	4,026	4,964	**0,041**	−3,477	5,139	0,077	−1,243	1,35	**0,025**
90	3,709	5,306	0,069	−3,486	4,909	0,066	−1,054	1,56	0,077

Fett gedruckte Zahlen zeigen einen signifikanten Unterschied (*p* < 0,05)

**Table 3:** 

	Mediolateral			Ventrodorsal			Proximodistal		
*Flexion (°)*	*MD*	*SD*	*p‑Wert*	*MD*	*SD*	*p‑Wert*	*MD*	*SD*	*p‑Wert*
0	7,91	4,393	**<** **0,001**	8,619	5,361	**0,001**	−2,529	4,104	0,102
10	7,888	4,833	**0,001**	7,858	5,046	**0,002**	−2,905	3,851	0,054
20	7,039	4,732	**0,002**	5,521	5,236	**0,013**	−2,358	3,496	0,078
30	6,718	5,014	**0,004**	3,676	5,688	0,089	−1,976	3,483	0,127
40	6,076	4,893	**0,006**	1,937	6,168	0,374	−1,589	3,37	0,195
50	5,061	4,732	**0,012**	−0,254	7,598	0,923	−1,278	3,349	0,285
60	4,749	4,56	**0,014**	−1,16	7,514	0,656	−0,967	3,206	0,392
70	4,118	4,508	**0,025**	6,114	13,245	0,203	−0,929	3,028	0,384
80	4,1	4,79	**0,033**	−2,227	6,648	0,344	−0,752	2,715	0,43
90	3,724	5,078	0,059	0,344	5,616	0,175	−0,623	2,899	0,537

Fett gedruckte Zahlen zeigen einen signifikanten Unterschied (*p* < 0,05)

## Diskussion

In der vorliegenden Studie findet sich zusammengefasst nach endoprothetischer Versorgung eine gute Rekonstruktion des nativen koronaren Alignments (Varus‑/Valgus) bei jedoch deutlich vermehrter tibialer Innenrotation in Streckung und 10° Beugung. Damit übereinstimmend findet sich der Epicondylus lateralis in Bezug zur Tibia nach KTEP-Implantation ebenfalls während der ersten 20° Beugung weiter dorsal als im nativen Kniegelenk. Der Epicondylus medialis präsentiert sich nach endoprothetischer Versorgung zwischen 40 und 80° Beugung etwas proximalisiert. Insgesamt zeigen sowohl der Epicondylus medialis als auch lateralis nahezu über das gesamte Bewegungsausmaß eine Translation nach lateral als Ausdruck einer relativen Translation des Femurs gegenüber der Tibia nach lateral. Zusammenfassend wird das Alignment des Kniegelenkes bzw. der unteren Extremität, wenn auch das koronare Alignment gleichbleibt, signifikant verändert.

Für die vorliegende Studie erfolgte die Implantatpositionierung über ein Navigationssystem. Dabei zeigt sich in der koronaren Ebene eine sehr exakte Rekonstruktion der Anatomie der gesunden Gelenke. Dies spiegelt auch die bekannte Literatur wider, wobei mit Navigationseinrichtungen wie auch Robotiksystemen per se eine sehr hohe Präzision erreicht werden kann. Durch die erhöhte Implantationsgenauigkeit konnte bislang allerdings nur unzureichend ein besseres klinisches Ergebnis für Patienten nachgewiesen werden. Lediglich in Langzeitstudien konnte eine geringere Revisionsrate nach navigationsgestützter KTEP-Implantation gezeigt werden [5]. So findet sich im australischen Register bei jungen Männern eine geringere Revisionsrate, wenn ein Navigationssystem bei der Implantation eines künstlichen Kniegelenkes zum Einsatz kommt [4].

Im vorliegenden Kollektiv zeigt sich, dass es nach KTEP-Implantation zwischen voller Streckung und 10° Beugung zu einer signifikant erhöhten Innenrotation des Unterschenkels gegenüber der des Femurs kommt. Dies ist durch die tibiale Komponentenaußenrotation zu erklären: Durch den relativen Formschluss der Femurkomponente und dem Inlay in voller Streckung, welche für die notwendige Stabilität während des Gangzyklus sorgt, führt eine Tibiakomponentenaußenrotation bekanntermaßen zu einer tibialen Innenrotation [14, 15]. Dieser Effekt der relativen Tuberositasmedialisierung kann zur Zentrierung der Kniescheibe verwendet werden. In vorausgegangenen Studien konnte ebenfalls gezeigt werden, dass die Rotation der Komponenten einen direkten Einfluss auf das femorale Rollback bzw. die tibiale Innenrotation haben [14, 15]. Bekannt ist auch, dass das koronare Komponentenalignment im Sinne eines „kinematischen Alignment“ direkten Einfluss auf das femorale Rollback bzw. die tibiale Innenrotation hat [16]. Nicht außer Acht gelassen werden sollte, dass aufgrund der Rotationsänderung zwischen Femur und Tibia auch der restliche Weichteilmantel um das Kniegelenk „torquiert“ werden kann. Weichteilschmerzen wären möglich Konsequenzen [17].

Hinsichtlich der Translationsparameter zeigt sich nach KTEP-Implantation sowohl für den Epicondylus medialis wie auch den Epicondylus lateralis eine Lateralisation nahezu über das gesamte Bewegungsausmaß. Dies bedeutet, dass das Femur in Relation zur Tibia nach lateral „shiftet“. Ein möglicher Erklärungsansatz ist die beschränkte Auswahl an Implantatgrößen und der damit verbundenen Möglichkeit, sowohl das tibiale wie auch das femorale Implantat mit einem gewissen Spielraum mediolateral auszurichten. Zudem läuft der Schienbeinkopf medial proximal nach distal konisch zu, sodass bei einer proximalen Tibiaresektion korrelierend mit dem Resektionsausmaß der mediale Rand zunehmend lateral zu liegen kommt. Um einen medialen Überhang der Komponente zu vermeiden, muss die Komponente nach lateral gesetzt werden. Auch die Femurkomponente kann üblicherweise in einem gewissen Ausmaß nach medial oder lateral versetzt werden. Aufgrund der Konformität zwischen Tibia bzw. tibialem Inlay und Femurimplantat hat das eine direkte Auswirkung auf die knöcherne Stellung zwischen Tibia und Femur [7].

In der vorliegenden Studie zeigt sich, dass nach KTEP-Implantation die laterale Epikondyle zwischen 0 und 20° Beugung signifikant dorsaler zur Tibia liegt als bei den Gelenken vor endoprothetischer Versorgung. Auch dies kann als Ausdruck der vermehrten tibialen Innenrotation interpretiert werden. Da das Rotationszentrum nicht streng zentral liegt, führt dies zu einer überwiegenden Beeinflussung des lateralen und weniger des medialen Kompartiments.

Zwischen 40 und 80° Beugung findet sich in unserem Kollektiv eine Proximalisierung des Epicondylus medialis. Dies kann als Folge der spezifischen Operationstechnik bzw. des Implantates gewertet werden. Während in Streckung und 90° Beugung die Gelenklinie üblicherweise gut rekonstruiert werden kann, besteht für die Beugegrade dazwischen eine für den Operateur bestehende Grauzone, die vom Radius der Endoprothese, der Extension und Flexion der Femurkomponente sowie vom tibialen Slope abhängt.

In der gesamten Diskussion darf nicht außer Acht gelassen werden, dass eine veränderte Position der Epikondylen zu einer Verschiebung der Bandansätze führt: Frei nach dem Gesetz nach Burmester bzw. der Logik der Bandisometrie stehen die Gelenkoberfläche und die Bandansätze in einem strengen geometrischen Zusammenhang zueinander: Veränderungen der Position der Epikondylen und damit der Bandansätze könnten Änderungen der Bandspannung im Sinne eines zu straffen Seitenbandes mit Bewegungseinschränkung, aber auch einer Laxizität des Seitenbandes mit (Midflexion‑)Instabilität des Gelenkes zur Folge haben [13]. Dasselbe ist für das hintere Kreuzband denkbar.

Die vorliegende Studie hat einige Limitationen. Es wurde lediglich ein Implantat und eine Implantationstechnik untersucht. Es ist davon auszugehen, dass unterschiedliche Implantate und Operationstechniken auch unterschiedliche Ergebnisse liefern. Das verwendete Navigationssystem liefert eine Genauigkeit von etwa 0,5–1,0° [10]. Die Untersuchungen wurden an unbelasteten Gelenken ohne den Einfluss des Muskelzugs durchgeführt. Wenn in MRT-Studien auch Unterschiede in der Kinematik zwischen belasteten und unbelasteten Kniegelenken bestehen, konnte gezeigt werden, dass diese Unterschiede nur sehr gering und vorhersehbar sind [18]. Der große Vorteil der vorliegenden Studie ist, dass mit nur einem Versuchsaufbau die Kinematik bzw. Stellung von Femur und Tibia vor und nach Implantation einer Endoprothese ohne neuerliche Registrierung untersucht werden kann. Ferner wurde die gesamte Extremität untersucht. Das gelingt in üblichen Kinematorstudien nicht.

Zusammenfassend zeigt sich in der vorliegenden Studie nach endoprothetischer Versorgung eine gute Rekonstruktion des koronaren Alignments. Hingegen kommt es im Rahmen der endoprothetischen Versorgung zwischen Femur und Tibia zu einer Veränderung des Alignments in rotatorischer und translatorischer Richtung. Mittels Navigation, aber auch Robotiksystemen, wären wir in der Lage, sämtliche Alignmentparameter zu quantifizieren und eine Ausrichtung der Komponenten bzw. die Rekonstruktion des Gesamtalignments in allen sechs Freiheitsgraden zu erzielen. Womöglich könnten wir dadurch auch einen klinischen Vorteil durch die Verwendung von Navigations- oder Robotiksystemen erzielen bzw. könnten die Standzeiten von Endoprothesen noch weiter erhöht werden.

## Fazit für die Praxis

- Navigations- und Robotiksysteme haben in der Endoprothetik eine sehr hohe Präzision hinsichtlich koronarer Endoprothesenausrichtung (Varus/Valgus) gezeigt.
- Je Komponente sind für die Implantation mit drei rotatorischen (Varus/Valgus, Rotation, Flexion bzw. Slope) und drei translatorischen Freiheitsgraden (mediolaterale Verschiebung, ventrodorsale Verschiebung, proximodistale Verschiebung) insgesamt sechs Freiheitsgrade in der Ausrichtung möglich.
- Mit der Navigation und der Robotik könnten wir sämtliche Ausrichtungsparameter quantifizieren und während der Implantation berücksichtigen.
- Dadurch könnten wir ggf. die klinischen Ergebnisse bzw. die Standzeiten von Kniegelenkendoprothesen noch weiter verbessern.

## Abkürzungen

CAS: computergestützte Assistenzsysteme
DOF: Freiheitsgrade
KTEP: Knietotalendoprothese
xf: x‑Achse Femur
xk: x‑Achse Kniegelenk
xt: x‑Achse Tibia
yf: y‑Achse Femur
yk: y‑Achse Kniegelenk
yt: y‑Achse Tibia
zf: z‑Achse Femur
zk: z‑Achse Kniegelenk
zt: z‑Achse Tibia
